# Predicting for Malignancy in Congenital Lung Malformations: A Single‐Center Study of the “At Risk” Pediatric and Adult Population

**DOI:** 10.1111/1759-7714.70410

**Published:** 2026-10-30

**Authors:** Medha Sridhar Rao, Eamonn Bradley, Alina Haddad, Suzanne Lawther, Alistair Dick, Gary Doherty, Clinton Boyd, Micheal Shields, Mary Patrice Eastwood

**Affiliations:** ^1^ School of Medicine, Dentistry and Biomedical Sciences Queen's University Belfast Belfast UK; ^2^ Department of Pediatric Surgery Royal Belfast Hospital for Sick Children, Belfast Health and Social Care Trust Belfast UK; ^3^ Pediatric Respiratory Medicine Unit Royal Belfast Hospital for Sick Children, Belfast Health and Social Care Trust Belfast UK; ^4^ Northern Ireland Regional Pathology Service Belfast Health and Social Care Trust Belfast UK

**Keywords:** congenital lung malformations, congenital pulmonary airway malformations, mucinous adenocarcinoma, pediatric lung malformations

## Abstract

**Introduction:**

Congenital lung malformations (CLMs) are often identified on antenatal scanning and malignant transformation is cited as an indication for early resection. Long‐term follow‐up of asymptomatic lesions managed nonoperatively is lacking. Herein, we prospectively follow up children with antenatally identified CLMs and reviewed adult patients diagnosed with adenocarcinoma to look for background CLM changes.

**Methods:**

This is a single‐center cohort study of pediatric patients with antenatal or early postnatal diagnosis of CLMs and a retrospective review of adult patients with lung adenocarcinoma. In children, data collected included type or location of CLMs and subsequent follow‐up. In adult patients, we reviewed radiology for background CLMs.

**Results:**

One hundred and twenty‐nine pediatric patients diagnosed with CLMs: congenital pulmonary airway malformations (*n* = 79; 61%), bronchopulmonary sequestrations (*n* = 26; 20%), congenital lobar emphysema (*n* = 11; 9%), resolved lesions (*n* = 10; 8%), bronchogenic cysts (*n* = 2; 2%), and an unknown lesion (*n* = 1; 1%). The patients were followed up for 6.7 (IQR: 3.4–10.5; range 0.08–20.6) years. No pediatric patient was subsequently identified on the Northern Ireland Cancer Registry. Three hundred and twenty‐three adult patients were diagnosed with lung carcinoma: 37 (11%) were mucinous adenocarcinomas and 21 (57%) had a KRAS mutation. No CLMs were identified on review of radiological imaging in adult patients presenting with malignancy.

**Conclusion:**

In children with CLMs, no resected malignant lesions could be identified on medium‐term follow‐up. Furthermore, no CLMs were identified in adult patients presenting with resected mucinous adenocarcinoma. Prospective collaborative databases are needed to determine true malignancy risk in asymptomatic CLM lesions and CPAMs.

## Graphical abstract

This single‐center outcome study of prospectively identified pediatric congenital lung malformations and concurrent retrospective incidence in adult mucinous carcinoma found that in children with CLMs no resected malignant lesions could be identified on medium‐term follow‐up. Furthermore, no CLMs could be identified in adult patients presenting with resected mucinous adenocarcinoma.

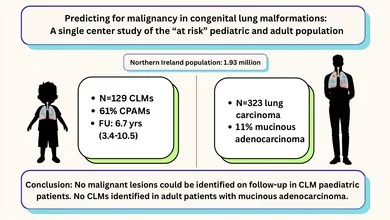

## 1 Introduction

Congenital lung malformations (CLMs) are a group of lesions often identified on antenatal scanning. They include congenital pulmonary airway malformations (CPAMs), bronchopulmonary sequestration (BPS), congenital lobar emphysema (CLE), and hybrid lesions [1]. CPAMs are CLMs that arise from the tracheobronchial tree [2]. In Europe, the prevalence of CPAMs is estimated to be 1.23 (1.17–1.29) out of 10 000 births [3]. The development of malignancy within CPAMs has particularly been associated with expression of the following genes: *dicer 1, ribonuclease III* (DICER‐1), *fibroblast growth factor receptor‐2* (FGFR2), *Kirsten rat sarcoma virus* (KRAS), and *tumor protein p53* (TP53) [4].

CPAMs can be diagnosed antenatally on ultrasound (US) scan or fetal magnetic resonance imaging (MRI) [2]. Fetal US identifies 75% of congenital lung lesions with others identified postnatally due to symptomatology [5]. Fetal MRI has the advantage of improved sensitivity for identifying feeding vessels into lesions but has similar sensitivity to US scanning [5, 6]. Postnatally, symptomatic lesions are often managed surgically; however, both surgical resection and nonoperative management of asymptomatic CPAMs have been reported [7]. One of the major concerns is the association between CPAMs and subsequent malignancy risk [8]. A recent systematic analysis review of adult patients presenting with symptomatic CPAMs suggested up to 24.1% of these had malignant change on resection [8]. Additionally, patients with a lung tumor were reported to be 3.2 times more likely to have CPAMs than patients with no lung tumor [8].

In published literature, the lung tumor type has been associated with the nature of the CPAM; primarily, if the lesion is microcystic or macrocystic [2]. Type I CPAMs have been associated with mucinous bronchoalveolar adenocarcinoma and KRAS mutations, whereas Type IV CPAMs are associated with pleuropulmonary blastomas (PPB) [9]. CPAMs have also been reported to be associated with several other lung tumors occurring later in the child's life [8].

There are several reported risk factors associated with the development of tumors in CPAMs [8]. A low malignancy rate is reported in asymptomatic CPAMs; reassuring features include the presence of systemic feeding vessels and DICER‐1 negative genetics [10]. Although postoperative complications following resection are low, bleeding requiring transfusion occurs in up to 14.8% of patients operated on between 1 and 3 months of age [11]. Furthermore, the conversion rate from thoracoscopy to thoracotomy during the resection of lung lesions is reported to be as high as 21.8% in children over 2 years of age [11]. Conversely, the nonoperative management of lesions requires life‐long radiological follow‐up [7, 12].

We have been offering conservative management for asymptomatic CPAMs in selected children since 1995 at our tertiary center. Herein, we review our cohort of children who have undergone prospective nonoperative management of CPAMs with longitudinal follow‐up. We checked for the presence of pediatric patients on the regional cancer registry to ascertain any development of malignant transformation. Furthermore, we retrospectively review a concurrent adult cohort presenting with adenocarcinoma to identify our rate of background CPAMs within an at‐risk population.

## 2 Methods and Materials

The study was approved by the Belfast Health and Social Care Trust and the Royal Belfast Hospital for Sick Children (RBHSC) as an Audit/Service Development (audit number 7162 and 6074). Research ethics approval was not required after consultation with the Health Research Authority Reporting Tool (Supplementary Material 1) [13].

### 2.1 Pediatric CLM Cohort

This was a single‐center cohort study of pediatric patients with CLMs from 1995 to 2023; patients with CPAMs were the main group analyzed in our study. Data collected included demographics, age of diagnosis, scanning modalities, symptomatology, indications for operative management, and duration of follow‐up when a conservative management approach was instigated. All included patients were searched for in the *Northern Ireland Cancer Registry* (NICR).

### 2.2 Retrospective Adult Lung Cancer Cohort

All adult patients diagnosed with adenocarcinoma and PPB from January 2021 to December 2024 were identified at our regional referral center.

Patients were identified based on biopsy results from the regional pathology service servers: *LabCenter* and *PathManager*. Search terms included adenocarcinoma ± mucinous adenocarcinoma (MAC) ± PPB. The data collected on patients included diagnosis, smoking status, symptomatology, imaging modalities, treatment (surgical, radiotherapy, and chemotherapy), and pathology (including genetic analysis of the tumor). Imaging and radiological reports (computerized tomography [CT], fluorodeoxyglucose positron emission tomography (FDG PET), chest X‐ray [CXR]) for all patients were reviewed for the presence of background CPAM lesions. Suspicious lesions were discussed with a second author (M.P.E.) and reviewed by radiology (PL) to confirm the presence or absence of a CLM in the adult population.

The following care records databases were used to collect data: *Northern Ireland Electronic Care Record* (NIECR), *Encompass* (NI EPIC), and the radiological images and reports were from the *Northern Ireland Picture and Communication System* (NIPACS).

The data analysis was performed using RStudio (GNU General Public License GPL Version 3) and GraphPad (2025‐GraphPad‐Software‐version Prism 10.6.0). Data were checked for normality and represented as median and interquartile range (IQR) or mean and standard deviation. The normality was checked using the Shapiro–Wilk test. Statistical significance was tested using a 2 × 2 Fisher's exact test.

## 3 Results

### 3.1 Pediatric Retrospective Cohort

At our pediatric center we identified 129 cases of CLMs from 1995 to 2023 (Figure 1): CPAMs (*n* = 79; 61%), BPS (*n* = 26; 20%), CLE (*n* = 11; 8%), BG cyst (*n* = 2; 2%) and an unknown lesion (*n* = 1; 1%). Ten (8%) patients were reported to have resolved CLMs on follow‐up CT scans (*n* = 6) or CXR (*n* = 4). Seventy‐two out of 129 (56%) patients were female and *n* = 114 (88%) CLMs were diagnosed antenatally. The follow‐up time for all lesions was a median of 6.7 (IQR: 3.4–10.5; range 0.08–20.6) years. The current ages of pediatric patients in this cohort ranged from 2 to 31 years old.

**FIGURE 1:**
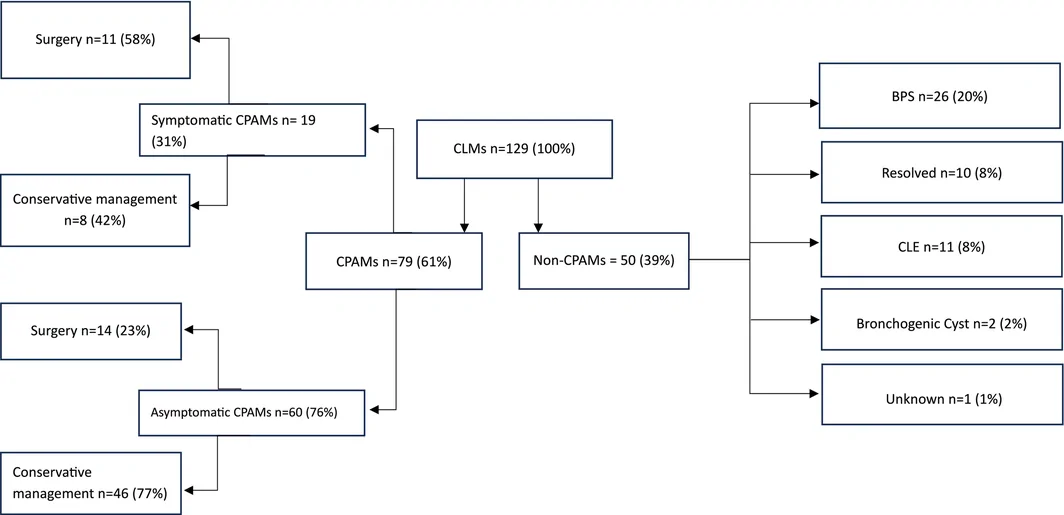
Flowchart of CLMs in our pediatric cohort.

### 3.2 Radiological Follow‐Up of CPAMs

Seventy‐nine pediatric patients had CPAMs: *n* = 72 (91%) patients were antenatally diagnosed; *n* = 7 (9%) were diagnosed coincidentally upon presentation to hospital for another reason or due to the presentation of postnatal symptoms. Fifteen (19%) were hybrid lesions. Sixty‐seven (85%) patients underwent postnatal CXRs, and of those who had a postnatal CXR, only *n* = 33 detected the presence of cystic lesions or CPAMs (Table 1). Twelve patients within the CPAM cohort had no postnatal CXRs, however, three of these patients had a CT at 9.8 ± 13.3 months of life (Table 1).

**TABLE 1:** Postnatal radiological investigations for CPAMs.

Radiological investigations	Cases (*n* = 79)	Age at CT examination (days)^b^	Years and months
Neonatal chest X‐ray^a^			
Yes	67 (85%)		
No	12 (15%)		
Number of CT scans performed^a^			
None	16 (20%)		
CT 1	64 (81%)	283 (424)	0 years and 9 months
CT 2	55 (70%)	1060 (823)	2 years and 10 months
CT 3	19 (24%)	1866 (1310)	5 years and 1 month
CT 4	9 (11%)	2530 (1459)	11 years and 11 months
CT 5	1 (1%)	5316 (1254)	14 years and 6 months

^a^ Number of cases (%).

^b^ Mean (standard deviation).

Sixty‐four (81%) patients had at least one CT (CT1); of these, some patients had further CT scans: CT2 (*n* = 55), CT3 (*n* = 19), CT4 (*n* = 9), and CT5 (*n* = 1) (Table 1). Fifteen patients (19%) did not have a CT scan. The age at the first CT was 4.5 (IQR 1–10.7) months (Table 1). CPAMs were most frequently identified in the right lung (*n* = 47; 59%), especially in the right lower lobe (*n* = 32; 40%). Symptomatic CPAMs (*n* = 19; 24%) were most frequently identified in the left upper lobe (*n* = 6; 8%). Asymptomatic CPAMs (*n* = 60; 76%) were most frequently seen in the right lower lobe (*n* = 28; 35%).

### 3.3 Management of Symptomatic CLMs (Non‐CPAMs)

Nine patients with non‐CPAM CLMs (BPS *n* = 2, CLE *n* = 5, BG cyst *n* = 2) were symptomatic: chest infections (*n* = 4), respiratory distress (*n* = 2), and unknown (*n* = 3). For three patients, the documentation of symptoms was inadequate on notes review. Eight patients underwent surgical resection of their symptomatic CLM. One symptomatic patient, with a documented chest infection in a CLE lesion, ultimately had expectant management, as no further infections were documented. The surgical approaches included: Total pneumonectomy (*n* = 1), lobectomy (access type not stated; *n* = 2), lobectomies through a thoracotomy (*n* = 3), cyst excision through thoracotomy (*n* = 1), and sequestration resection (*n* = 1). Complications included pectus excavatum (*n* = 1), air leak from the left main bronchus (*n* = 1), and pneumothoraces (*n* = 2). These patients were followed up for a median of 6.3 (IQR: 2.9–11.6, range: 0.5–18.1) years (Table 2).

**TABLE 2:** Clinical characteristics and management of symptomatic congenital lung malformations (CLMs).

	Overall (*n* = 28)	CPAM (*n* = 19)	Non‐CPAM (*n* = 9)
Symptoms			
Chest infections^a^	7 (25%)	3 (16%)	4 (44%)
Respiratory distress^a^	4 (14%)	2 (11%)	2 (22%)
Operative approach^b^	19 (68%)	11 (58%)	8 (89%)
Lobectomy	14 (50%)	9 (47%)	5 (56%)
Segmentectomy	0	0	0
Cyst excision	2 (7%)	1 (5%)	1 (11%)
Other resections	2 (7%)	0	2 (22%)
Awaiting surgery	1 (4%)	1 (5%)	
Complications^c^	9 (32%)	5 (26%)	4 (44%)
Conservative management	9 (32%)	8 (42%)	1 (11%)
Histology			
Type I CPAM	2 (7%)	2 (11%)	
Type II CPAM	0	0	
Unknown CPAM type	8 (28%)	8 (42%)	

^a^ Additional symptoms: CPAM: cyst location (*n* = 1; 5%), cyst size (*n* = 1; 5%), unknown (*n* = 1; 5%). Non‐CPAM: unknown (*n* = 3; 33%).

^b^ Operative approach: CPAM (*n* = 11)—lobectomy: thoracotomy (*n* = 6; 32%), thoracoscopy (*n* = 1; 5%), unknown (*n* = 2; 11%); cyst excision: thoracotomy (*n* = 1; 5%). Non‐CPAM (*n* = 7): lobectomy: thoracotomy (*n* = 3; 33%), unknown (*n* = 1; 11%); pneumonectomy: (*n* = 1; 11%); cyst excision (thoracotomy *n* = 1; 11%); thoracoscopic sequestration resection (*n* = 1; 11%).

^c^ Complications: CPAM: pneumothorax (*n* = 4; 21%), pectus excavatum (*n* = 1; 5%). Non‐CPAM: pneumothorax (*n* = 2; 22%), air leak (*n* = 1; 11%), pectus excavatum (*n* = 1; 11%).

### 3.4 Management of Symptomatic CPAMs

Nineteen (24%) patients had symptomatic CPAMs (Figure 1); 10 patients required surgery for symptomatic management and one patient, included in the chest infection group, is currently awaiting surgery. Indications for surgery at the time of surgical booking included: respiratory compromise (*n* = 5), chest infections (*n* = 3), cyst location (*n* = 1), cyst size (*n* = 1), unknown (*n* = 1). Nine patients underwent lobectomies and one patient had a cyst excision via thoracotomy. The approach for lobectomies included thoracotomy (*n* = 6), thoracoscopy (*n* = 1), and an unspecified approach (*n* = 2). The mean age at surgery was 1.9 ± 3.1 years. Postoperatively, five patients had complications which included pneumothoraces (*n* = 4) and pectus excavatum (*n* = 1) (Table 2).

The histopathology analysis reported Type I CPAMs (*n* = 2) and the data for the remaining eight patients was unavailable.

Eight patients continue to be managed nonoperatively as they are clinically stable.

The median follow‐up time was 5.5 (IQR: 2.8–9.0; range: 0.08–19.5) years.

### 3.5 Management of Asymptomatic CLMs (Non‐CPAMs)

Forty (31%) patients had asymptomatic non‐CPAM CLMs: BPS *n* = 24, CLE *n* = 6 and resolved CLMs *n* = 10. Of these, five required surgery. Indications for surgery included: pulmonary hypertension (*n* = 1), lobar inflation (*n* = 1), and unknown (*n* = 3). The surgical approaches were: Two lobectomies (approach unknown), one partial lobectomy via thoracotomy, one total pneumonectomy (approach unknown) and one thoracoscopic resection of a sequestration. One patient developed pectus excavatum postoperatively. Thirty‐five patients were managed conservatively owing to stable or resolving radiological features. The group with resolved CLMs did not have any surgical interventions or complications from the disease. The median follow‐up time was 6.7 (IQR: 3.2–11.1; range: 0.5–20.6) years (Table 3).

**TABLE 3:** Clinical characteristics and management of asymptomatic congenital lung malformations (CLMs).

	Overall (*n* = 100)	CPAM (*n* = 60)	Non‐CPAM (*n* = 40)
Indications for surgery			
Radiological concerns	3 (3%)	3 (5%)	
Mass effect/mediastinal shift	2 (2%)	2 (3%)	
Other indications^a^	13 (13%)	8 (13%)	5 (13%)
Operative approach	19 (19%)	14 (23%)	5 (13%)
Lobectomy	10 (10%)	8 (13%)	2 (5%)
Segmentectomy	1 (1%)	1 (8%)	
Cyst excision	0	0	
Other resections^b^	8 (8%)	5 (38%)	3 (8%)
Awaiting surgery	1 (1%)	1 (2%)	
Complications^c^	3 (3%)	2 (3%)	1 (3%)
Conservative management	81 (81%)	46 (77%)	35 (88%)
Histology			
Type I CPAM	4 (4%)	4 (7%)	
Type II CPAM	3 (3%)	3 (5%)	
Unknown	6 (6%)	6 (10%)	

^a^ Indications for surgery—CPAM: unknown (*n* = 8; 13%). Non‐CPAM: pulmonary hypertension (*n* = 1; 3%), lobar inflation (*n* = 1; 3%) and unknown (*n* = 3; 8%).

^b^ Operative approach—CPAM: lobectomy: thoracotomy (*n* = 4; 7%), thoracoscopy (*n* = 2; 3%), unknown (*n* = 2; 3%), converted (*n* = 1; 2%), partial lobectomy (*n* = 1; 2%); segmentectomy (converted) (*n* = 1; 2%) and unknown approach (*n* = 2; 3%). Non‐CPAM: lobectomy (*n* = 2; 5%), partial lobectomy (thoracotomy) (*n* = 1; 3%), pneumonectomy (unknown approach) (*n* = 1; 3%) and sequestration resection (thoracoscopic) (*n* = 1; 3%).

^c^ Complications: CPAM: pneumothorax (*n* = 1; 2%) and surgical emphysema (*n* = 1; 2%). Non‐CPAM: pectus excavatum (*n* = 1; 3%).

### 3.6 Management of Asymptomatic CPAMs

Sixty (47%) patients had asymptomatic CPAMs. Of the 60 asymptomatic patients, 46 patients were conservatively followed up as they remained clinically stable. However, 13 patients, initially managed conservatively, had surgical management at a mean age of 19.1 ± 15.9 months. One (2%) patient has been referred to our center's adult thoracic surgical team. Therefore, 14 patients were or will be surgically managed (Table 3).

The indications for surgery (*n* = 13) included: mass effect (*n* = 1), mediastinal shift (*n* = 1), radiological concerns (*n* = 3) and unknown (*n* = 8). The operative methods included: partial lobectomy (unknown approach; *n* = 1), lobectomy (unknown approach; *n* = 2), lobectomy with thoracotomy (*n* = 4), a segmentectomy converted from thoracoscopy to thoracotomy (*n* = 1), a thoracoscopic lobectomy (*n* = 2), a lobectomy converted from thoracoscopy to thoracotomy (*n* = 1) and unknown surgical method (*n* = 2) (Table 3). Two patients experienced postoperative complications: pneumothorax (*n* = 1) and surgical emphysema (*n* = 1) (Table 3).

The histological analysis of the 13 resected CPAMs showed results for seven patients: Type I CPAMs (*n* = 4) and Type II CPAMs (*n* = 3). The results of CPAM classification were unavailable for six patients (Table 3).

The median follow‐up time for this group was 7.7 years (IQR: 4.5–10.6 years and range: 0.2–20.6 years).

No patient with a CLM was identified on the NICR.

### 3.7 Adult Retrospective Patient Demographics

We identified *n* = 323 adult patients with lung adenocarcinoma. No adult patients were identified with PPB in this timeframe.

Adenocarcinoma was diagnosed at a median age of 69 years old (IQR: 62–75). The majority of patients were female (*n* = 179; 56%) and over four‐fifths of patients had a current or previous smoking history (*n* = 277; 86%). No patients in our cohort were prenatally diagnosed with CPAMs (Table 4).

**TABLE 4:** Adult cohort demographics.

	Overall	Mucinous	Nonmucinous	*p*
Number of patients	323 (100%)	37 (11%)	286 (89%)	
Male/female	Male: 144 (45%) Female: 179 (55%)	Male: 16 (43%) Female: 21 (57%)		
Smoker	277 (86%)	26 (70%)	251 (88%)	0.009817^c^
Alive	214 (66%)	29 (78%)	185 (65%)	0.1382
Prenatal diagnosis	0 (0%)	0 (0%)	0 (0%)	
Symptomatology				0.01638^c^
Incidental	68 (21%)	14 (38%)	54 (19%)	
Cough	104 (32%)	10 (27%)	94 (33%)	0.5763
Sputum	25 (8%)	4 (11%)	21 (7%)	0.5081
Weight loss	24 (7%)	1 (3%)	23 (8%)	0.3344
SOB	83 (26%)	7 (19%)	76 (27%)	0.4241
Hemoptysis	23 (7%)	1 (3%)	22 (8%)	0.4933
Recurrent infections	78 (24%)	8 (22%)	70 (25%)	0.8392
Other symptomatology	42 (13%)	5 (14%)^a^	37 (13%)^b^	1.0

^a^ Wheeze = 1, hypercalcemia = 1, MSK weakness/pain = 2, lobar collapse = 1.

^b^ MSK/pleuritic pain.

^c^ Significant results.

### 3.8 Adult Cohort: Symptomatology

Overall, patients commonly experienced cough (*n* = 104; 32%) with excessive sputum production *n* = 25 (8%) or hemoptysis in *n* = 23 (7%). Dyspnea was present in *n* = 83 (27%) and recurrent infections in *n* = 78 (24%). Over one‐fifth of patients had no symptoms, and the tumor was found incidentally on CT scanning (*n* = 68; 21%) (Table 4). Thirty‐seven patients had mucinous adenocarcinoma where the most common presentation was cough (*n* = 10; 27%) (Table 4). The number of smokers was higher in the nonmucinous group (*p* < 0.05), and the presence of symptomatology was higher in the mucinous adenocarcinoma group (*p* < 0.05) (Table 4).

### 3.9 Pathology

Overall, *n* = 402 specimens were analyzed by our pathology unit. Core needle biopsies (CNB) (*n* = 181; 56%) and lymph node biopsies (*n* = 92; 28%) were the most frequently analyzed specimens. In the mucinous adenocarcinoma group (*n* = 37), lepidic subtypes (*n* = 27; 73%) were the most frequently seen followed by acinar in *n* = 13 (35%) patients (Table ^5^).

**TABLE 5:** Genetic and histological analysis of the lung cancer resections.

Genetics	Mucinous	Nonmucinous	*p*
KRAS	21 (57%)	54 (19%)	< 0.0001
DICER‐1	0 (0%)	0 (0%)	
Other mutations	7 (19%)^a^	164 (57%)^b^	< 0.0001
No mutations	8 (22%)	90 (32%)	0.2579
Histological subtypes			
Lepidic	27 (73.0%)	68 (24%)	< 0.0001
Acinar	13 (35%)	59 (21%)	0.05815
Papillary	0 (0%)	38 (13%)	
Micropapillary	4 (10%)	20 (7%)	0.4996
Solid	1 (3%)	48 (17%)	0.0257
Unknown	0 (0%)	75 (26%)	
Management			0.4617
Nonoperative	10 (27%)	97 (34%)	
Operative	27 (73%)	189 (66%)	
Unknown	0 (0%)	0 (0%)	

^a^ ERBB2: 2 (5.4%), MET: 1 (2.7%), PD‐L1: 4 (10.4%).

^b^ ERBB2: 3 (1.0%), MET: 8 (2.8%), PD‐L1: 96 (33.6%), EGFR: 43 (15.0%), ROS: 1 (0.3%), RET: 1 (0.3%), BRAF: 8 (2.8%), ALK: 4 (1.4%).

### 3.10 Mutations Present in the Adult Population

The resected specimens were analyzed using tumor genetic analysis. A KRAS mutation was present in *n* = 75 (23%); *n* = 57 (18%) of patients having KRAS‐only mutations and *n* = 18 (6%) had KRAS mutations in combination with other mutations. No resected adult lung specimens underwent a DICER‐1 genetic test. The KRAS mutation was higher in the mucinous group (*p* < 0.0001) whereas other mutations were higher in the nonmucinous group (*p* < 0.0001) (Table 5). Additionally, lepidic and solid histological patterns were significantly higher in the mucinous group (*p* < 0.05) (Table 5).

### 3.11 Adult Cohort: Radiological Findings

FDG PET was used for staging in 34 (91.9%) patients in the mucinous adenocarcinoma group and in 250 (87.4%) patients in the nonmucinous group (Table 6).

**TABLE 6:** Radiological findings in the adult cohort.

	Mucinous	Nonmucinous
Computed tomography (CT)	3 (8.1%)	36 (12.6%)
FDG PET	34 (91.9%)	250 (87.4%)
Pathology identified on imaging		
CPAMs identified	0 (0%)	0 (0%)
Cavitating lesions	6 (16.2%)	33 (11.5%)

Six (16.2%) patients in the mucinous adenocarcinoma group and 33 (11.5%) patients in the nonmucinous group had cavitating lesions; however, no background CPAMs could be identified on any preoperative scans (Table 6).

Overall, no background CPAM was identified in our adult population on histological or radiological analysis.

## 4 Discussion

We report no malignancy in our pediatric cohort with CLMs followed up from birth until the age of 7 years (range: 0.08–20.6 years). Furthermore, in a 3‐year adult cohort of pulmonary adenocarcinoma, we report no CPAMs on radiological imaging at the time of diagnosis.

In our pediatric cohort, the right lower lobe was the most common location for CPAMs (41%), with symptomatic CPAMs being most commonly identified in the left upper lobe (32%). Adult CPAMs are commonly reported in the left lower lobe followed by the right lower lobe [8]. However, the most common site for adenocarcinoma in our adult cohort was the right upper lobe (31%) followed by the left upper lobe (23%). High percentages of adult adenocarcinoma (53%) have been reported previously in the upper lobes [14, 15]. These discrepancies in the anatomical distribution of CPAMs and the adenocarcinoma may suggest different pathologies. Adenocarcinoma without a background of CPAMs is more likely to be located in the upper lobes and adenocarcinoma with a background of CPAMs is more likely to be present in the lower lobes.

In Pederiva et al., 25.6% of adult patients (39.6 ± 16.0 years) with lung tumors had background CPAMs; the most common malignancy was adenocarcinoma [8]. These tumors were primarily diagnosed due to symptomatology (74%); 6% were smokers and the authors reviewed patients' CT scans to identify the background CPAM. In asymptomatic patients, an incidental diagnosis of adenocarcinoma was made during preoperative workups for unrelated conditions or routine health check‐ups. The presence of symptomatology was significantly higher in adult patients in the mucinous adenocarcinoma group (*p* < 0.05).

In our adult population (323 patients) diagnosed with lung adenocarcinoma, the average age of diagnosis was 69 years old (range 42–89) and 86% were smokers. Our population is older and more likely to have a history of smoking than the population reported in the systematic review [8]. Casagrande and Pederiva reported the age of diagnosis of adenocarcinoma associated with CPAM was younger at mean of 53.1 ± 17.5 years [16]. Smoking was a strong contributing factor for the formation of adenocarcinoma in our population; 86% were current smokers or had a history of smoking. The number of patients who had a history of smoking in the nonmucinous population was significantly higher than in the mucinous population (*p* < 0.05). In the systematic review's population, 21.2% of the people with CPAMs had a history of smoking before the development of an adenocarcinoma [8]. The prevalence of smoking is around 14% in the Northern Ireland population which is slightly lower compared with the global figure of 15% [17]. It is possible the lower smoking rates in Northern Ireland may have an impact on the risk of malignant transformation in patients with CPAMs [17, 18].

We could not identify any radiological evidence of background CPAMs in patients with adenocarcinoma, including the 30 patients diagnosed with mucinous adenocarcinoma. However, if we apply the incidence rates from Casagrande and Pederiva, we would have predicted eight patients with background CPAMs [8]. Variations in our population, including genetics and lower smoking rates, could have a role in this difference [8].

The management of asymptomatic CLMs in pediatric patients is controversial. The main concern is the risk of malignancy developing within these lesions [8]. Single centers have reported high complication rates (23%) following asymptomatic CPAM resection at around 6 months including bleeding, air leaks, and conversion from thoracoscopy to open thoracotomy [7]. However, some report early resection to be associated with shorter operating times in patients at 1–3 months [19]. A UK‐based survey (2013) of pediatric surgeons found that only 21% would routinely resect asymptomatic lesions [7]. At our center, the mean age at resection for asymptomatic CPAMs was 19.1 ± 15.9 months with a complication rate of 15%. The indications for surgery in these patients included the size of the lesion (> 2.5 cm from 2005 to 2015 and > 3 cm from 2015) or mass effect of the lesion in the thorax, the perceived risk of potential infections or cysts acutely increasing in size during air travel. Most of the patients who were at risk of these features would have elective surgery prior to starting school. Additionally, we documented only two conversions from thoracoscopic surgery to a thoracotomy at an average age of 28.8 months which is in keeping with the literature [11]. For other CLMs, a tailored approach was adopted. In CLE, if the affected lung lobe was overinflated and collapsing the residual lobe or causing mediastinal shift, elective surgical lobectomy was recommended. In a BPS with a large vessel which had shunting, the procedure of choice was an embolization of the vessel. The European Delphi Study (2022) analyzed the management of CPAMs from 28 centers across Europe [20]. It is stated that 98% of surgeons surveyed rated respiratory insufficiency as a strong factor for the consideration of surgery [20]. Mass effect was an indication for resection in babies (< 1 month) and children (> 3 months) [20].

At our center, all pediatric CLMs are discussed at thoracic multidisciplinary team meetings involving pediatric surgeons, respiratory pediatric physicians, and pediatric radiologists. Over the last decade, our management of congenital pulmonary lesions has evolved [21]. Our center has adopted a more conservative and less surgical approach to asymptomatic CLMs post‐2010: Surgical management was 29% pre‐2010 and 13% post‐2010 [21]. Typically, children with asymptomatic low‐risk lesions (such as the presence of a feeding vessel or antenatal diagnosis) are offered the option of nonoperative management at our center. These children have a postnatal CXR and a CT scan on at least one, and usually two occasions. The first CT is usually between six and 12 months of age. A second CT scan for larger or more worrying lesions is carried out at the age of 5 years. A third CT may be discussed before the transition to adult service. The development of symptomatology prompts expedited imaging and multidisciplinary discussion regarding surgical excision.

Our center conducts genetic analysis on pathology specimens in adult patients suspected of having a lung malignancy: five genes are checked for mutations, including KRAS, which is associated with CPAMs‐related malignancy [2, 4]. Muntean et al. reported an association between neonatally diagnosed symptomatic Type I CPAMs containing mucinous adenocarcinoma and a KRAS mutation [22]. Hermelijn et al. found KRAS mutations to be present in every CPAM sample containing mucinous proliferation and within some nonmucinous cells from CPAM samples; normal lung tissue near the CPAM was found to have mucinous and nonmucinous cells without a KRAS mutation [23]. In our adult population, mucinous adenocarcinoma had significantly higher KRAS mutations (*p* < 0.05) without an associated CPAM. Therefore, when considering tissue genetic screening for our pediatric patients with CPAMs, the KRAS gene mutation testing might be a useful addition to the genetic screening panel.

Our study has several limitations. Despite prospective follow‐up, the adult and pediatric cohorts from our center are small. Additionally, our identified CPAMs were not routinely classified by type. Advances in antenatal imaging have resulted in the identification of CLMs that would previously not have been recognized. Therefore, the population of adults presenting with adenocarcinoma does not have longitudinal follow‐up of lung lesions had they existed prenatally. We do not routinely offer DICER‐1 genetics to asymptomatic pediatric CPAM patients; therefore, we may be at risk of missing some high‐risk patients. In our adult cohort, we reviewed imaging at the time of cancer diagnosis and any preceding thoracic imaging: We did not identify any CPAMs, and the distortion of the anatomy may have limited our ability to recognize preexisting background CPAMs. The lack of longitudinal follow‐up limits our ability to comment on the preexisting CLM existence. Furthermore, we identified patients from positive histology specimens; we do not have any adult patients with incidentally identified CPAMs. We also do not routinely offer systemic genetic analysis, and tumor analysis is the only way to gather genetic data in the adult patient cohort.

## 5 Conclusion

We did not identify the development of any malignant lesions, including mucinous adenocarcinoma or PPB, in pediatric patients with asymptomatic CLMs followed up prospectively. Furthermore, we could not identify any background CPAMs in adult patients presenting with adenocarcinoma in our population.

At our center, we remain reluctant to offer major surgery to all children with asymptomatic CLMs solely on the basis of potential malignancy in adulthood, as the evidence supporting this practice is, at best, equivocal. Our unit was reassured by our follow‐up of conservatively managed CLMs and in the absence of clinical standard guidelines, we disseminate our findings. Prospective longitudinal collaborative databases are needed to determine true malignancy risk in asymptomatic CLM lesions.

## Author Contributions

**Medha Sridhar Rao:** conceptualization, data curation, writing – original draft, formal analysis, visualization, supervision, methodology, investigation, project administration, writing – review and editing, software, validation, resources. **Eamonn Bradley:** conceptualization, data curation, formal analysis, visualization, writing – review and editing, project administration, methodology, investigation, supervision, software, validation, resources. **Alina Haddad:** conceptualization, data curation, formal analysis, visualization, methodology, investigation, supervision, project administration, writing – review and editing, software, validation, resources. **Suzanne Lawther:** conceptualization, data curation, formal analysis, visualization, methodology, investigation, supervision, project administration, writing – review and editing, software, validation, resources. **Alistair Dick:** conceptualization, data curation, visualization, formal analysis, methodology, investigation, supervision, project administration, writing – review and editing, software, validation, resources. **Gary Doherty:** conceptualization, data curation, formal analysis, visualization, writing – original draft, methodology, investigation, project administration, supervision, writing – review and editing, software, validation, resources. **Clinton Boyd:** conceptualization, data curation, formal analysis, visualization, methodology, project administration, investigation, supervision, writing – review and editing, software, validation, resources. **Micheal Shields:** conceptualization, data curation, formal analysis, visualization, writing – original draft, methodology, investigation, supervision, project administration, writing – review and editing, software, validation, resources. **Mary Patrice Eastwood:** conceptualization, data curation, formal analysis, visualization, writing – original draft, methodology, investigation, supervision, writing – review and editing, project administration, software, validation, resources.

## Funding

The authors have nothing to report.

## Ethics Statement

Research ethics approval was not required for this study after consulting the Health Research Authority Reporting Tool (Supplementary Material 1) [13].

## Conflicts of Interest

The authors declare no conflicts of interest.

## Supporting information


**Supplementary Material 1:** HRA Reporting Tool.

## Data Availability

The data are not publicly available due to privacy or ethical restrictions.
