# Supplementary material for: Predicting for Malignancy in Congenital Lung Malformations: A Single‐Center Study of the “At Risk” Pediatric and Adult Population
Source: Thorac Cancer. 2026 Oct 30;17(21):e70410. doi: 10.1111/1759-7714.70410 (PMC13722533; doi:10.1111/1759-7714.70410)
Supplement: Supplementary file 1 [file TCA-17-e70410-s001.docx]

Supplementary Material 1: HRA Reporting Tool
